# One New and Nine Known Flavonoids from *Choerospondias axillaries* and Their *in Vitro* Antitumor, Anti-Hypoxia and Antibacterial Activities

**DOI:** 10.3390/molecules191221363

**Published:** 2014-12-19

**Authors:** Chang-Wei Li, Cheng-Bin Cui

**Affiliations:** State Key Laboratory of Toxicology and Medical Countermeasures, Beijing Institute of Pharmacology and Toxicology, Beijing 100850, China; E-Mail: sdrlcw@126.com

**Keywords:** *Choerospondias axillaries*, isolation, flavonoids, antitumor, anti-hypoxia, antibacterial

## Abstract

In the present study, a new flavanoid **1**, together with nine known ones **2**–**10** were isolated from the stem bark of *Choerospondias axillaries*, the fruit of which was used mainly for treatment of cardiovascular diseases in China. The structure of **1** was established on the basis of its extensive spectral data, and the absolute structures of **1** and **10** were determined by their CD data. The absolute structure of **10** was established for the first time. Among the obtained compounds, **5**–**8** inhibited the proliferation of K562 cells with inhibition rates of 26.6%, 65.7%, 40.4% and 45.6% at 100 µg/mL; **1** and **4**–**10** showed significant protective effects on anoxia-induced injury in cultured ECV304 or PC12 cells at 50 µg/mL; **8** and **9** showed antibacterial effects on *Staphylococcus aureus* ATCC6538 at the tested concentration of 150 µg/8 mm paper disc. Compounds **2** and **4**–**10** were isolated for the first time from this genus. The proliferation inhibiting activities of **7** and **8**, the anti-hypoxia activities of **1** and **4**–**10**, and the antibacterial effect of **8** and **9** on *Staphylococcus aureus* ATCC6538 are reported here for the first time.

## 1. Introduction

**Figure 1:**
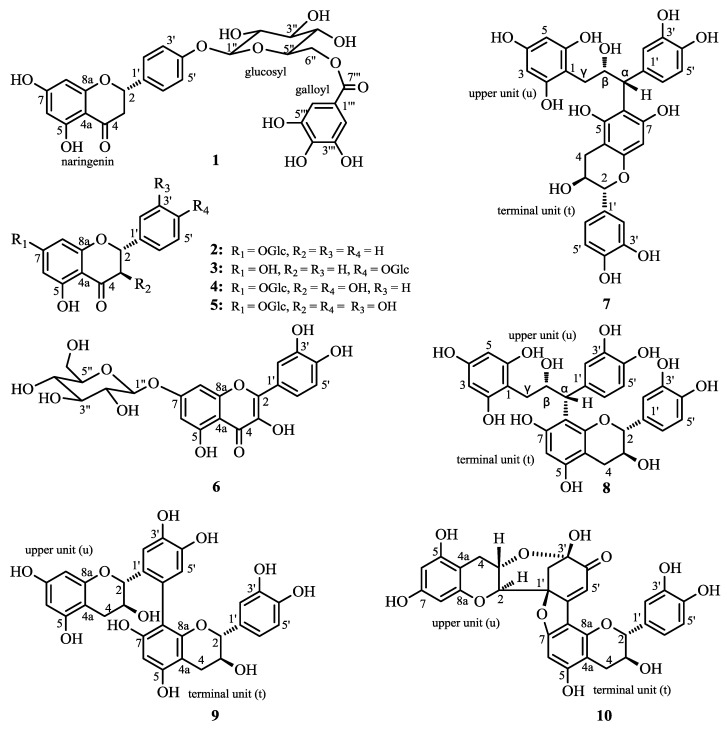
Structures of compounds **1**–**10**.

*Choerospondias axillaries*, the only plant of the genus *Choerospondias* belonging to the family of Anacardiaceae, is mainly distributed in the Hubei, Guangdong, Guangxi, Yunnan, Fujian and Guizhou provinces of China. In China, the dried fruit of *C. axillaries* has been usually used for the treatment of cardiovascular diseases, especially in Mongolian traditional medicine [1,2]. Chemical studies of the plant have revealed the presence of phenolic compounds, flavonoids, sterols, organic acids and polysaccharides, and the total flavonoids were always assumed to be the effective constituents behind its medicinal usage [2]. Our previous work on screening herbal medicines for antitumor activity showed that the ethanol extract of the stem bark of *C. axillaries* exhibited strong cytotoxicity [3,4]. An ongoing study of its bioactive constituents has now led to isolation of one new and nine known flavonoids **1**–**10** (Figure 1). In this work, the isolation, identification, the absolute structure determination and bioactivities of the ten flavonoids **1**–**10** are reported.

## 2. Results and Discussion

### 2.1. Structure Elucidation of **1** and Identification of **2**–**10**

**Figure 2:**
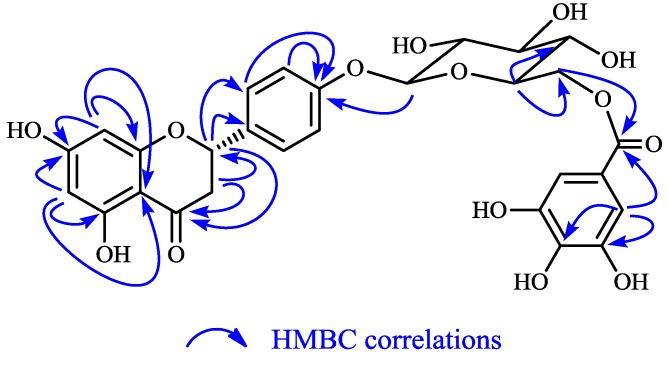
Structure and the HMBC correlations of **1**.

Compound **1** was obtained as white needle-like crystals, m.p. 160–162 °C (from 30% methanol/water solution), ${\left[\alpha \right]}_{D}^{25}$ −18.9° (c 1.0, MeOH), and showed a dark blue coloration upon spraying with FeCl_3_ reagent on the silica gel TLC plate. Positive ion HR-ESI-MS showed a quasi-molecular ion peak at *m/z* 587.14907 [M+H]^+^, and adduct peaks at 609.12290 [M+Na]^+^ and 625.09327 [M+K]^+^ corresponding to a formula of C_28_H_26_O_14_. Its UV spectrum showed a maximum absorption at 286 nm (log ε, 4.03) arising from the hydroxylated phenyl groups. Its IR spectrum displayed characteristic absorptions for hydroxyl (3293 cm^−1^) and carbonyls (1685 and 1637 cm^−1^). The ^1^H-NMR spectrum showed aliphatic proton signals at δ 3.07 (1H, dd, *J* = 16.8, 12.8 Hz) and at δ 2.66 (1H, dd, *J* = 16.8, 2.8 Hz) from the axial and equatorial methylene protons at C-3 as well as a third aliphatic resonance at δ 5.31 (1H, dd, *J* = 12.8, 2.8 Hz) from the methine proton, which bears an oxygen and phenyl function at C-2 of the flavanone moiety. The AA′BB′ system at δ 7.29 (2H, d, *J* = 8.4 Hz) and at δ 7.06 (2H, d, *J* = 8.4 Hz), and an AX system at δ 5.90 (1H, d, *J* = 2.0 Hz) and at δ 5.88 (1H, d, *J* = 2.0 Hz) were in accordance with derivatives of 5,7,4′-trihydroxyflavonone, *i.e.*, derivatives of naringenin. The anomeric proton signal at δ 4.91 (1H, d, *J* = 7.6 Hz) revealed the presence of one sugar with a β configuration. The ^13^C-NMR signals at δ 101.5, 77.5, 75.1, 74.3, 71.5 and 64.4 indicated that the sugar should be glucosyl group [5], so except for the additional signals [δ_C_: 167.9, 146.0 (2C), 139.4, 120.8, 109.8 (2C)] arising from a galloyl group, the ^13^C-NMR data of **1** were closely similar to those of naringenin-4′-*O*-β-d-glucopyranoside (**3**) (Table 1). The linkage of the glucopyranosyl moiety to the 4′-hydroxyl group of naringenin was confirmed by the HMBC correlation between the anomeric proton at δ 4.91 (1H, d, *J* = 7.6 Hz) of the glucopyranosyl moiety and C-4′ at δ 158.5 (Figure 2). The location of the galloyl was determined to be the glucose C-6″ position according to the HMBC correlations between the glucose 6″-H and carbonyl carbon of galloyl group (Figure 2), which was also supported by the downfield shift of glucose 6″-H at δ 4.38 (1H, dd, *J* = 8.4, 12.0 Hz) and 4.61 (1H, dd, *J* = 2.0, 12.0 Hz). Thus, compound **1** was identified as narigenin-4′-*O*-(6″-*O*-galloyl-β-D-glucopyranoside).

**Table 1:** 400 MHz ^1^H-NMR and 100 MHz ^13^C-NMR data of **1** and **3** ^a^.

Position	1 ( in CD_3_OD)		3 ( in DMSO- *d*_6_)	
	δ_H_	δ_C_	δ_H_	δ_C_
narigenin				
2	5.31 (1H, dd, *J* = 12.8, 2.8 Hz)	79.7	5.53 (1H, dd, *J* = 12.4, 3.0 Hz)	78.6
3	3.07 (1H, dd, *J* = 16.8, 12.8 Hz)2.66 (1H, dd, *J* = 16.8, 2.8 Hz)	43.3	Overlapped 2.74 (1H, dd, *J* = 17.2, 3.0 Hz)	42.5
4		197.3		196.7
4a		102.9		102.3
5		164.9		163.3
6	5.90 (1H, d, *J* = 2.0 Hz)	96.6	5.90 (1H, d, *J* = 2.4 Hz)	96.4
7		167.6		167.2
8	5.88 (1H, d, *J* = 2.0 Hz)	95.8	5.89 (1H, d, *J* = 2.4 Hz)	95.5
8a		164.3		164.0
1′		133.5		132.4
2′	7.29 (1H, d, *J* = 8.4 Hz)	128.4	7.44 (2H, d, *J* = 8.6 Hz)	128.6
3′	7.06 (1H, d, *J* = 8.4 Hz)	117.1	7.07 (2H, d, *J* = 8.6 Hz)	116.7
4′		158.5		158.1
5′	7.06 (1H, d, *J* = 8.4 Hz)	117.1	7.07 (2H, d, *J* = 8.6 Hz)	116.7
6′	7.29 (1H, d, *J* = 8.4 Hz)	128.4	7.44 (2H, d, *J* = 8.6 Hz)	128.6
5-OH			12.13 (1H, s)	
7-OH			10.82 (1H, s)	
glucosyl				
1″	4.91 (1H, d, *J* = 7.6 Hz)	101.5	4.89 (1H, d, *J* = 7.6 Hz)	100.8
2″	3.49–3.54 (1H, m)	74.3	3.13–3.49 (1H, m)	73.7
3″	3.49–3.54 (1H, m)	77.5	3.13–3.49 (1H, m)	77.6
4″	3.42 (1H, m)	71.5	3.13–3.49 (1H, m)	70.2
5″	3.78 (1H, m)	75.1	3.13–3.49 (1H, m)	77.1
6″	4.38 (1H, dd, *J* = 12.0, 8.4 Hz)4.61 (1H, dd, *J* = 12.0, 2.0 Hz)	64.4	3.13–3.49 (1H, m) 3.70 (1H, dd, *J* = 10.6, 4.0 Hz)	61.2
galloyl				
1‴		120.8		
2‴	7.10 (1H, s)	109.8		
3‴		146.0		
4‴		139.4		
5‴		146.0		
6‴	7.10 (1H, s)	109.8		
7‴ (C=O)		167.9		

^a^ The δ_H_ and δ_C_ values were recorded using solvent signals (CD_3_OD: δ_H_ 3.31/δ_C_ 49.0 for **1**; DMSO-*d*_6_: δ_H_ 2.50/δ_C_ 39.5 for **3**) as references. Signal assignments were based on the results of ^1^H–^1^H COSY, HMQC and HMBC experiments.

**Figure 3:**
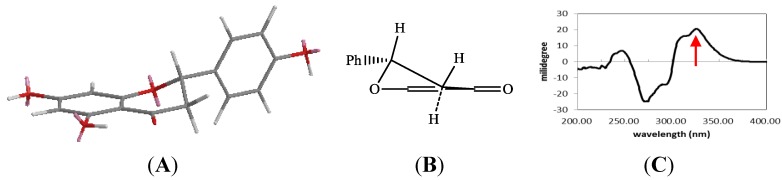
The CD spectrum and the absolute structure of **1**. (**A**): The absolute structure of **1**; (**B**): The conformation of **1**; (**C**): CD spectrum of **1** in MeOH (1 mg/mL).

The absolute 2*S* structure of narigenin was confirmed by the positive Cotton effect at 326.5 nm in the CD spectrum [6] (Figure 3). Analogous structures of **1** had been reported before [5], but the 6″-galloyl-glucopyranosyl moiety was connected to C-7 of the naringenin aglycone.

Compound **10** was obtained as a yellow crystalline powder, m.p. 196–198 °C (MeOH), ${\left[\alpha \right]}_{D}^{24}$ −332.7° (c 0.5, MeOH), also showing a dark blue coloration with FeCl_3_ reagent on silica gel TLC. The ^1^H-NMR and ^13^C-NMR data of compound **10** were consistent with the data reported for dehydrodicatechin A [7], with an as yet undetermined absolute structure (Table 2).

**Table 2:** 400 MHz ^1^H-NMR and 100 MHz ^13^C-NMR data of **10** in CD_3_OD ^a^.

Position	δ_H_	^1^H-^1^H COSY	δ*_C_*	HMBC (H→C)			NOESY
2(u)	3.96 (1H, m)		78.1	C-3(u), 4(u), 6′(u)			4(u)-Hb
3(u)	3.96 (1H, m)	4(u)-H	65.5	C-2(u), 4(u), 1′(u)			4(u)-Ha, 2′(u)-Ha
4(u)	Ha:2.52 (1H, dd, *J* = 9.0,14.4 Hz) Hb:2.92 (1H, dd, *J* = 5.8,14.4 Hz)	3(u)-H 3(u)-H	27.0	C-2(u), 3(u), 4a(u), 5(u)			3(u), 4(u)-Hb 2(u), 4(u)-Ha
4a(u)			99.1				
5(u)			155.0 ^c^				
6(u)	5.52 (1H, d, *J* = 2.6 Hz) ^b^	8(u)	94.4 ^d^	C-4a(u), 5(u),7(u), 8(u)			
7(u)			156.4 ^c^				
8(u)	5.89 (1H, d, *J* = 2.6 Hz) ^b^	6(u)	95.7 ^d^	C-4a(u), 6(u), 7(u), 8a(u)			
8a(u)			156.7 ^c^				
1′(u)			88.5				
2′(u)	Ha:2.67 (1H, d, *J* = 11.6 Hz) Hb:2.48 (1H, d, *J* = 11.6 Hz)		44.3	C-2(u),1′(u),3′(u),6′(u)			3(u), 2′(u)-Hb 2′(u)-Ha
3′(u)			94.0				
4′(u)			192.8				
5′(u)	6.41 (1H, s)		111.5	C-2′(u),1′(u),3′(u),6′(u)			
6′(u)			162.8				
2(t)	4.92 (1H, d, *J* = 7.5Hz)	3(t)-H	82.1	C-3(t), 4(t), 1′(t), 2′(t),6′(t),8a(t)			4(t)-Ha
3(t)	4.11 (1H, td, *J* = 7.5,5.2 Hz)	2(t)-H, 4(t)-H	66.5	C-4a(t)			4(t)-Hb
4(t)	Ha:2.59 (1H, dd, *J* = 7.5,16.4 Hz) Hb:2.85 (1H, dd, *J* = 5.2,16.4 Hz)	3(t)-H 3(t)-H	26.5	C-2(t), 3(t), 4a(t), 5(t), 8a(t)			2(t), 4(t)-Hb 3(t), 4(t)-Ha
4a(t)			102.6				
5(t)			164.9				
6(t)	6.11 (1H, s)		89.6	C-5(t), 8(t)			
7(t)			166.7				
8(t)			104.3				
8a(t)			153.8				
1′(t)			129.9				
2′(t)	6.84 (1H, d, *J* = 2.2 Hz)	5′(t)	113.5	C-2(t), 4′(t), 6′(t)			2(t), 3(t)
3′(t)			145.0 ^e^				
4′(t)			145.2 ^e^				
5′(t)	6.78 (1H, d, *J* = 8.0 Hz)	2′(t), 6′(t)	115.0	C-1′(t), 3′(t)			
6′(t)	6.73 (1H, dd, *J* =2.2,8.0 Hz)	5′(t)	118.4	C-2(t), 2′(t), 4′(t)			2(t), 3(t)

^a^ The δ_H_ and δ_C_ values were recorded using solvent signals (CD_3_OD: δ_H_ 3.31/δ_C_ 49.0) as references. Signal assignments were based on the results of ^1^H–^1^H COSY, HMQC and HMBC experiments signals. ^b–e^ The signals could not be assigned exactly.

**Scheme 1:**
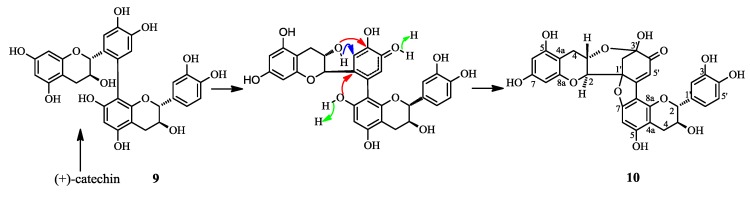
The plausible biosynthetic pathway of compound **10**.

**Figure 4:**
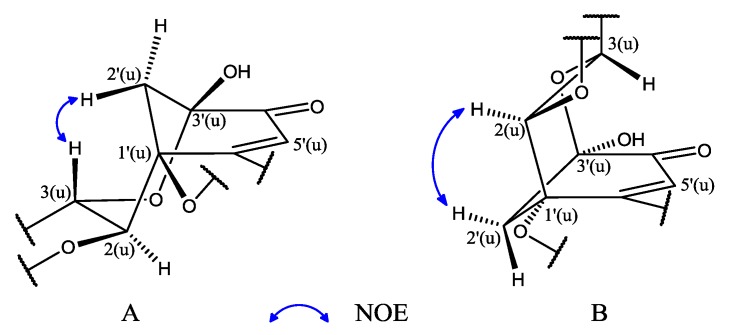

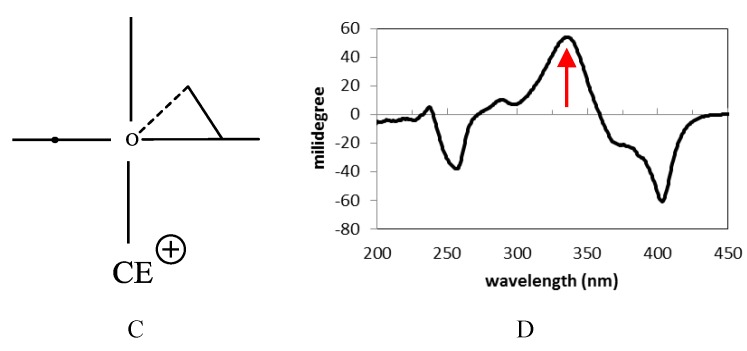
(**A**,**B**) the two conformations of compound **10**; (**C**) Standard conformation of cyclohexenone ring showing the positive CE and its application of the octant rule; (**D**) The CD spectrum of compound **10** (1 mg/mL, MeOH).

In the literature compound **10** was first obtained as an oxidation product of (+)-catechin [7] and in this work we also detected this compound among the products resulting from heating (+)-catechin, so the sterochemistry of **10** should be identical to that of (+)-catechin, except for the absolute configurations of C-1′(u) and C-3′(u) (Scheme 1). In the NOE experiment, 2′(u)-Ha showed correlation with 2(u) or 3(u)-H which were overlapped, so there were two conformations for this compound, conformation A or B (Figure 4). To determine the absolute structure of **10**, the CD spectrum was obtained, which showed a positive Cotton effect at 336.5 nm (n–π* transition). According to the helicity rule [8,9], the conformation of **10** should be A, and the absolute configuration of B ring should be 1′(u)*S* and 3′(u)*S*, so the complete absolute structure of **10** was determined. Though two reports [10,11] have described the stereochemistry of **10**, both of them ultimately referred to reference [7] which did not establish the absolute structure of **10**, so this paper reports for the first time the whole stereochemistry of **10**.

Compounds **2**–**5** were all obtained as white crystalline powders, and compound **6** was obtained as yellow needle-like crystals. On the basis of their ESI-MS, ^1^H-NMR and ^13^C-NMR spectral data, compounds **2**–**6** were identified as pinocembrin-7-*O*-β-d-glucopyranoside (**2**) [12], naringenin-4′-*O*-β-d-glucopyranoside (**3**) [13], dihydrokaempferol-7-*O*-β-d-glucopyranoside (**4**) [14], dihydroquercetin-7-*O*-β-d-glucopyranoside (**5**) [14] and quercetin-7-*O*-β-d-glucopyranoside (**6**) [15], respectively. Compounds **7** and **8**, obtained as crystalline powders, were identified as gambiriin A3 (**7**) [16] and gambiriin A1 (**8**) [17] according to the corresponding ESI-MS, ^1^H-NMR and ^13^C-NMR spectral data. The main characteristic of the structures of these two compounds is that the C ring of the upper unit is cleaved, which is rare in Nature. Compound **9** was identified as (+)-catechin (6′-8) (+)-catechin [7], which was also first obtained from among the oxidation products of (+)-catechin in the literature [7].

### 2.2. Biological Activities of **1**–**10**

#### 2.2.1. Antitumor Activity

**Figure 5:**
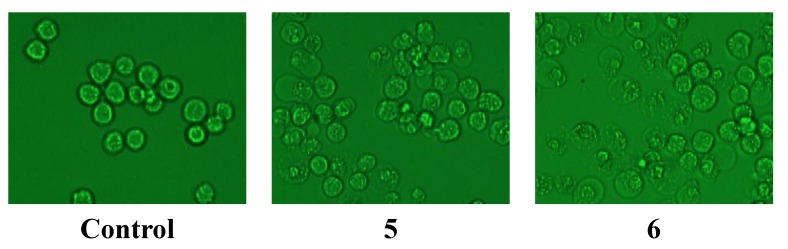

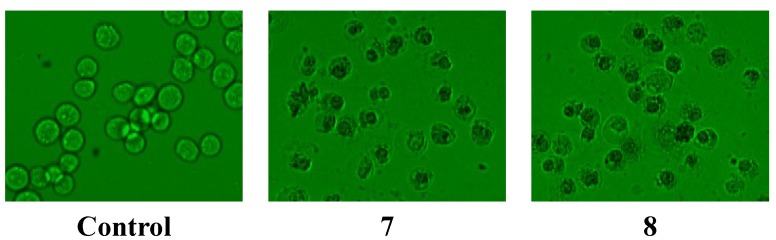
Photographs of K562 cells treated with **5**–**8** for 24 h at 100 µg/mL.

The antitumor activities of **1**–**10** on the K562 cells were evaluated by the MTT method [9], complemented by morphological observations of the cells under a light microscope. The proliferation-inhibiting effect on K562 cells of compounds **5**–**8** were detected by an MTT assay, which gave inhibition rates of 26.6%, 65.7%, 40.4% and 45.6% at 100 µg/mL, and the IC_50_ value of **6** is 60.7 µg/mL. The other six compounds showed no noticeable inhibition rates on K562 cells, *i.e.*, 3.4% for **1**, 3.9% for **2**, −0.5% for **3**, 10.4% for **4**, −39.8% for **9** and −16.2% for **10**, respectively. The morphology of the cells treated with **1**–**4**, **9** and **10** at 100 µg/mL for 24 h showed no distinction from the control group, but inflated cell membranes and cell content leakage could be seen of the cells treated with **5**–**8** which showed apparent cytotoxicity on K562 cells (Figure 5). The positive control docetaxol inhibited the K562 cells with an IR% value of 57.8% at 100 µg/mL.

#### 2.2.2. Anti-Hypoxia Effects

To evaluate the anti-hypoxia activities, ECV304 cells were used for **2**–**5** and PC12 cells were used for **1**, **6**–**10** as the tested cells by the MTT method. The MTT assays showed that **1**–**10** presented no cytotoxicity or inhibitory effect on ECV304 or PC12 cells, but the cell viabilities of the above two cell lines treated with **1** and **4**–**10** were notably increased at 50 µg/mL (Table 3), which suggested that **1** and **4**–**10** exhibited good anti-hypoxia activities.

**Table 3:** Anti-hypoxia effects of **1** and **4**–**10** on anoxic tested cells (50 µg/mL).

Samples	Tested Cells	Cell Viabilities (mean value ± SD%, n = 10)	
		Control Group	Test Group
**1**	PC12	90.0 ± 6.1	131.4 ± 15.3 **
**2**	ECV304	22.6 ± 0.1	17.1 ± 1.2
**3**	ECV304	22.6 ± 0.1	27.8 ± 1.5
**4**	ECV304	36.1 ± 1.7	46.4 ± 1.0 *
**5**	ECV304	36.1 ± 1.7	51.9 ± 0.9 ***
**6**	PC12	82.8 ± 5.3	108.1 ± 6.5 **
**7**	PC12	85.9 ± 4.0	174.0 ± 8.7 **
**8**	PC12	85.9 ± 4.0	155.6 ± 14.4 **
**9**	PC12	90.0 ± 6.1	120.8 ± 9.8 **
**10**	PC12	90.0 ± 6.1	122.7 ± 7.1 **

* *P* < 0.05, ** *P* < 0.01, *** *P* < 0.001, compared with model group.

#### 2.2.3. Anti-Bacterial Activity

The anti-bacterial activities for **1**–**10** were assayed by the 8 mm paper disc method using *Blastomyces albicans* ATCC10231 and S*taphylococcus aureus* ATCC6538. Compounds **8** and **9** inhibited the growth of S*taphylococcus aureus* ATCC6538 with 15 mm and 14 mm inhibition zones at the concentration of 150 µg/paper disc, respectively.

### 2.3. Discussion

Flavonoids are widely distributed in numerous plants, and have received considerable attention due to their diverse bioactivities [18,19]. Flavonoids with galloyl glucopyranosyl groups were also discovered in large numbers of plants [5,20,21,22,23,24,25]. To our knowledge, except for quercetin-4′-*O*-(6″-*O*-galloyl-β-D-glucopyranoside) [25], the galloylglucopyranosyl groups of all these compounds was connected to the A or C ring of the flavonoid skeleton. Compound **1** is the second example whose galloylglucopyranosyl was connected to the B ring of the flavonoid skeleton, which is very rare in Nature. Dimeric flavans, especially catechin derivatives, are reported as being isolated both from plants [10,11,16,17,26] and the oxidation products of catechin or epicatechin [7,27], and their biosynthesis and genetic regulation have also been investigated [28]. Dehydrodicatechin A (**10**), a dimeric flavan from (+)-catechin, was also isolated both from plants or among the oxidation products of (+)-catechin. Though the planar structure of **10** had been determined years ago, the exact absolute structure of this compound had not yet been established [7,10,11,27,29], so this paper, represents the first time that the absolute structure of **10** was established on the basis of its CD data.

## 3. Experimental Section

### 3.1. General Experiment Procedures

Melting point was measured on a Beijing Tiandiyu X-4 exact micro melting point apparatus (Tiandiyu Science and Technology Co., Ltd., Beijing, China) and the temperatures are not corrected. Optical rotations were measured on an Optical Activity Limited polAAr 3005 spectropolarimeter (Optical Activity Limited, Ramsey, UK). ESIMS was recorded on an Applied Biosystems API 3000 LC-MS spectrometer (AB SCIEX, Framingham, MA, USA) and HRESIMS was measured on an Agilent 6520 Q-TOF LC-MS spectrometer (Agilent Technologies, Santa Clara, CA, USA). IR spectra were taken on a Bruker Tensor-27 infrared spectrophotometer (Bruker, Karlsruhe, Germany). CD data were recorded on a Biologic Science MOS450 CD spectropolarimeter (Bio-Logic, Pont-de-Claix, France). 1D and 2D NMR spectra were obtained on a JEOL JNM-GX 400 (400 MHz ^1^H and 100 MHz ^13^C-NMR) NMR spectrometer (JASCO electric Co., Ltd., Tokyo, Japan). The chemical shifts of ^1^H and ^13^C NMR signals were recorded in δ values using the solvent signals (CD_3_OD: δ_H_ 3.31/δ_C_ 49.0; DMSO-*d*_6_: δ_H_ 2.50/δ_C_ 39.5) as references, respectively.

Precoated analytical silica gel GF_254_ plates (10 cm × 20 cm, 0.25 mm thickness, Yantai Chemical Industrial Institute, Yantai, China) and polyamide thin layers (10 cm × 20 cm, Taizhou Luqiao Sijia Biochemical Plastic Factory, Taizhou, China) were used in TLC and spots were detected under sunlight and UV light (254 and 365 nm) or by using Vaughan’s reagent (24 g of ammonium molybdate tetrahydrate (NH_4_)_6_Mo_7_O_24_·4H_2_O) and 1 g of ceric sulfate Ce(SO_4_)_2_ dissolved in 500 mL of 10% H_2_SO_4_) or 5% FeCl_3_ reagent (5 g of FeCl_3_ dissolved in 100 mL of 95% aqueous EtOH). Silica gel H (100–200 mesh, Yantai Chemical Industrial Institute), YMC*GEL^®^ ODS-A-HG (12 nm S-50 µm, YMC Co., Ltd., Kyoto, Japan), Sephadex™ LH-20 (GE Healthcare, Uppsala, Sweden), and polyamide (100–200 mesh, Taizhou Luqiao Sijia Biochemical Plastic Factory) were used for column chromatography.

Human chronic myelogenous leukemia K562 cell line was provided by Prof. Dr. Song Li (Beijing Institute of Pharmacology and Toxicology, Beijing, China). Fetal bovine serum was purchased from Tianjin Hao Yang Biological Manufacture Co., Ltd. (Tianjin, China). The RPMI-1640 medium (lot No. 0803238) was purchased from Gibco (Grant Island, NY, USA) and MTT (lot No. 0793) from Amresco (Solon, OH, USA). Streptomycin (lot No. 071104) and penicillin (lot No. X0803302) were purchased from North China Pharmaceutical Group Corporation, Beijing, China. Docetaxol (DOC, lot No.20080215) was purchased from Beijing Chimivo Technology Co., Ltd. (Beijing, China).

### 3.2. Plant Material

The stem bark of *C. axillaries* was collected in the Mengla region of Yunnan, China. The plant was identified by Professor Sun Qi-shi and a voucher specimen (No. 050901) was deposited at the Beijing Institute of Pharmacology and Toxicology.

### 3.3. Extraction and Isolation

The stem barks of *C. axillaries* (3.2 kg) was exhaustively extracted with 95% ethanol (25 L, 4 × 7 d) and 60% ethanol (25 L, 3 × 7 d) in turn to give 750 g and 90 g extracts, respectively. The 95% ethanol extract was further extracted from water with different organic solvents (3 L) to give CHCl_3_ extract (60 g), EtOAc extract (310 g) and *n*-BuOH extract (300 g).

The CHCl_3_ extract was subjected to silica gel column chromatography (bed: 7.5 × 18.5 cm) eluted with petroleum (P)–acetone (A)–methanol (M) solvent system, and the methanol eluate (12 g) was then further subjected to silica gel column chromatography and Sephadex LH-20 column chromatography to obtain **2** (300 mg) and **3** (180 mg).

The EtOAc extract (100 g) was subjected to polyamide column chromatography (bed: 7.0 × 50.0 cm) eluted with EtOAc–MeOH (Et:M 1:0–0:1) to obtain four fractions, E-1 (4 g, EtOAc eluate), E-2 (35 g, Et:M 9:1 eluate), E-3 (25 g, Et:M 1:1 eluate) and E-4 (15 g, MeOH eluate). E-3 was further subjected to polyamide chromatography (bed: 3.5 × 50.0 cm) eluted with CHCl_3_ (C)–CH_3_OH (M) (4:1), the main fraction containing **1** was recrystallized in 30% methanol/water solution to get **1** (517 mg). E-4 (15 g, MeOH eluate) was subjected to polyamide chromatography (bed: 3.8 × 50.0 cm) using a CHCl_3_–CH_3_OH gradient as eluting solvent, to give two fractions, E-41 and E-42. E-42 (12.0 g, MeOH eluate) was further separated by Sephadex LH-20 and polyamide column chromatography to obtain **7** (102 mg) and **8** (113 mg).

The *n*-BuOH extract was subjected to macroporous resin AB-8 column chromatography (bed: 8.5 × 48.0 cm) to get a water eluate (38 g). The water eluate was subjected to polyamide column chromatography (bed: 7.5 × 18.5 cm) eluted with a water (W)–acetone (A) gradient to give eight fractions, B-1 (10 g, water eluate), B-2 (2.0 g, water eluate), B-3 (1.5 g, W:A 9:1 eluate), B-4 (1.7 g, W:A 9:1 eluate), B-5 ( 2.0 g, W:A 9:1), B-6 ( 5 g, W:A 7:3 eluate), B-7 (5 g, W:A 5:5 eluate), B-8 ( 5 g, W:A 2:8 eluate). B-2 was separated by Sephadex LH-20 column chromatography (bed: 2.8 × 27.0 cm) with stepwise water–methanol (W–M) elution to obtain three fractions, B-21–B-23. Fraction B-22 (1.4 g, W:M 7:3 eluate) was further subjected to Sephadex LH-20 column chromatography (bed: 2.8 × 60.0 cm) eluted with 10% methanol to get **4** (35 mg) and **5** (520 mg). B-23 (200 mg, methanol eluate) was also separated by Sephadex LH-20 column chromatography (bed: 2.8 × 60.0 cm) eluted with 80% methanol to obtain **6** (26 mg).

The 60% ethanol extract was subjected to polyamide column chromatography (bed: 8.5 × 22.0 cm), eluted with gradient water (W)–ethanol (E)–acetone (A) to give five fractions, 60-1 (10 g, water eluate), 60-2 (17 g, water eluate), 60-3 (2 g, W:E 3:1 eluate), 60-4 (15 g, ethanol eluate) and 60-5 (20 g, acetone eluate). Fraction 60-4 was further subjected to polyamide column chromatography (bed: 4.5 × 50.0 cm) eluted with an EtOAc (Et)–MeOH (M) gradient to give seven fractions, 60-41–60-47. 60-46 (0.7 g, Et:M 15:1 eluate) was separated by Sephadex LH-20 column chromatography (bed: 1.5 × 70.0 cm) eluted with chloroform–methanol (1:1) to obtain **9** (37 mg). 60-47 (4.5 g, methanol eluate) was applied to polyamide and Sephadex LH-20 column chromatography to obtain **10** (105 mg).

### 3.4. Physicochemical Properties and Spectra Data

*Narigenin-4′-O-(6″-O-galloyl-β-D-glucopyranoside* (**1**). White crystalline powder, m.p. 160–162 °C (MeOH), ${\left[\alpha \right]}_{D}^{25}$ −18.9° (c 1.0, MeOH), showing a dark blue coloration with ferric chloride reagent. Positive ion ESI-MS *m/z*: 587 [M+H]^+^, 609 [M+Na]^+^, 625 [M+K]^+^. Positive ion HR-ESI-MS *m/z*: 587.14907 [M+H]^+^, 609.12290 [M+Na]^+^, 625.09327 [M+K]^+^. UV (MeOH) λ_max_ (log ε): 216 nm (4.41), 286 nm (4.03). IR (KBr) ν_max_: 3293, 2947, 2882, 1685, 1637, 1607, 1517, 1460, 1343, 1316, 1230, 1188, 1161, 1065, 1038 864, 744, 723 cm^−1^. CD λ_max_ nm (mdeg) in MeOH at 1.0 mg/mL: 234.0 (0), 244 (6.4100), 255.5 (0), 272.5 (−25.0052), 301.5 (0), 326.5 (20.5810), 383.0 (0). ^1^H-NMR and ^13^C-NMR data see Table 1.

*Naringenin-4′-O-β-D-glucopyranoside* (**3**). White crystaline powder, m.p. 224–226 °C (MeOH), ${\left[\alpha \right]}_{D}^{25}$ −49.5° (c 0.2, Me_2_CO), showing a brown with ferric chloride reagent. Positive ion ESI-MS* m/z*: 435 [M+H]^+^, 457 [M+Na]^+^, 473 [M+K]^+^. ^1^H-NMR and ^13^C-NMR data see Table 1.

*Dehydrodicatechin A* (**10**). Yellow crystalline powder, m.p. 196–198 °C (MeOH), ${\left[\alpha \right]}_{D}^{25}$ −332.7° (c 0.5, MeOH), showing a dark blue coloration with ferric chloride reagent. Positive ion ESI-MS *m/z*: 577 [M+H]^+^, 599 [M+Na]^+^; Negative ion ESI-MS *m/z*: 575 [M−H]^−^. CD λ_max_ nm (mdeg) in MeOH at 1.0 mg/mL: 190 (−0.3533), 232.5 (0), 237.5 (5.1513), 240 (0), 256.5 (−38.1595), 272.0 (0), 336.5 (53.8359), 358.5 (0), 403.0 (−60.7813), 456.0 (0). ^1^H-NMR and ^13^C-NMR data see Table 2.

### 3.5. Bioassays

#### 3.5.1. Cell Line and Cell Culture

Human myeloid leukemia K562, human umbilical vein endothelial cell ECV304, and rat pheochromocytoma PC12 cell lines were used for bioassay. The K562, ECV304 and PC12 cells were routinely maintained in RPMI-1640 (for K562 and ECV 304 cells) or DMEM (for PC12 cells) medium containing 100 µg/mL penicillin and 100 µg/mL streptomycin supplemented with 10% FBS under a humidified atmosphere of 5% CO_2_ and 95% air.

#### 3.5.2. Cell Proliferation Assay

Compounds **1**–**10** and DOC were dissolved in MeOH to prepare a 10.0 mg/mL solution, and serial dilutions were made for compounds **1**–**10**. These solutions were subjected to the MTT assay. DOC was used as positive control and MeOH was used as blank control. The assay was run in triplicate on human cancer K562 cell lines by the method that we have previously reported [9].

#### 3.5.3. Anti-Hypoxia Assay

Compounds **1**–**10** were dissolved in the DMEM or RPMI 1640 medium to prepare a solution at 50.0 µg/mL. The anti-hypoxia activities of **2**–**5** were assayed using ECV304 cells, but the anti-hypoxia activities of **1** and **6**–**10** were assayed on PC12 cells.

*MTT assay on ECV 304 cells*: Exponentially growing ECV 304 cells were suspended in fresh RPMI 1640 medium at the density of 1 × 10^5^ cells/mL and then seeded into 96-well plates at 150 µL/well. The cells were incubated at 37 °C for 48 h, then discarded the medium and were assigned into normoxic control group, hypoxia control group and hypoxia administration group. Each well of the normoxic control group and hypoxia control group was added 150 µL fresh RPMI 1640 medium, and the hypoxia administration group was 150 µL sample solution. The hypoxia control group and hypoxia administration group were cultured in the atmosphere of 5% CO_2_ and 95% N_2_ for 24 h, while the normal control group was normally cultured for 24 h. Then, 15 µL MTT solution (5 mg/mL in PBS) was added to each well and incubated at 37 °C for 4 h. Then, the MTT solution were discarded, and 150 µL DMSO were added in each well; after the purple material were fully dissolved, the optical density (OD) of each well was determined on a Versa max plate reader at 490 nm. The cell viabilities were calculated using mean values from cell viabilities (%) = OD _hypoxia control_ or OD _sample_/OD _normal control_ × 100%.

*MTT assay on PC 12 cells*: Exponentially growing PC12 cells were suspended in fresh DMEM medium at the density of 1 × 10^5^ cells/mL and then seeded into 96-well plates at 150 µL/well. The cells were incubated at 37 °C for 24 h, then discarded the medium and were assigned into normoxic control group, hypoxia control group and hypoxia administration group. Each well of the normoxic control group and hypoxia control group was added 150 µL fresh DMEM medium, and the hypoxia administration group was 150 µL sample solution, then all were incubated at 37 °C for 1 h. Each well of hypoxia control group and hypoxia administration group were added 2 µL CoCl_2_ solution (306.14 µg/mL in water), all the three groups were incubated at 37 °C. After all were normally incubated for 24 h, 15 µL MTT solution (5 mg/mL in PBS) was added to each well and incubated at 37 °C for 4 h. Then, the MTT solution were discarded, and 150 µL DMSO were added in each well; after the purple material were fully dissolved, the optical density (OD) of each well was determined on a Versa max plate reader at 490 nm. The cell viabilities were calculated using mean values from cell viabilities (%) = OD _hypoxia control_ or OD _sample_/OD _normal control_ × 100%.

#### 3.5.4. Antibacterial Effect Test

*Blastomyces albicans* ATCC10231 and S*taphylococcus aureus* ATCC6538 were used to evaluate the antibacterial activities of compounds **1**–**10**. *Blastomyces albicans* ATCC10231 was cultivated on Sabouraud’s agar medium, and S*taphylococcus aureus* ATCC6538 was cultivated on tryptose soya agar medium. All the compounds were dissolved in methanol to prepare 10 mg/mL test sample solutions. Samples (15 µL) were added to the paper discs (8 mm) and dried for 10 min. Then, the discs were put on the tested plate which contained either S*taphylococcus aureus* or *Blastomyces albicans*. Tested plates were cultured at 28 °C for 2 days and then the diameter of the inhibition zone was measured.

## 4. Conclusions

Ten flavonoids were isolated from the stem bark of *C. axillaries*. Among the obtained compounds, **1** is a new compound with a galloylglucosyl group on the B ring in the flavonoid skeleton, and compounds **2** and **4**–**10** were isolated from this genus for the first time. The absolute structures of **1** and **10** were established from their CD data, and the absolute configuration of **10** was determined exactly for the first time. The proliferation inhibiting activities of **7** and **8**, the anti-hypoxia activities of **1** and **4**–**10**, and the antibacterial effect of **8** and **9** on *Staphylococcus aureus* ATCC6538 are reported here for the first time.

## Supplementary Materials

Supplementary materials can be accessed at: http://www.mdpi.com/1420-3049/19/12/21363/s1.
