# Supplementary material for: One New and Nine Known Flavonoids from Choerospondias axillaries and Their in Vitro Antitumor, Anti-Hypoxia and Antibacterial Activities
Source: Molecules. 2014 Dec 19;19(12):21363–77. doi: 10.3390/molecules191221363 (PMC6271225; doi:10.3390/molecules191221363)

Supplementary Materials

Physicochemical and Spectroscopic Data of Seven Known Compounds 2 and 4–9:

Pinocembrin-7-O-β-D-glucopyranoside (**2**): White crystalline powder (MeOH), m.p. 130–132 °C, $[\alpha]_D^{25}$ −78.5° (c 0.2, Me_2CO), showing a brown coloration with ferric chloride reagent. Positive ion ESI-MS *m/z*: 419 $[M+H]^+$, 441 $[M+Na]^+$, 457 $[M+K]^+$; ^{1}H-NMR (400 MHz, DMSO-d_6) δ: 12.04 (1H, s, 5-OH), 7.53 (2H, d, *J* = 6.8 Hz, 2′, 6′-H), 7.43 (3H, m, 3′-H~5′-H), 6.20 (1H, d, *J* = 2.0 Hz, 6-H), 6.16 (1H, d, *J* = 2.0 Hz, 8-H), 5.65 (1H, dd, *J* = 12.8, 3.4 Hz, 2-H), 4.98 (1H, d, *J* = 7.6 Hz, 1″-H), 3.13-3.60 (6H, m, 2″-H~6″-H, 3-*H-trans* was overlapped), 2.85 (1H, dd, *J* = 17.6, 3.4 Hz, 3-H-*cis*); ^{13}C-NMR (100 MHz, DMSO-d_6) δ: 197.3 (C-4), 165.9 (C-7), 163.5 (C-8a), 163.1 (C-5), 139.0 (C-1′), 129.2 (C-3′, 4′, 5′), 127.3 (C-2′, 6′), 103.8 (C-4a), 100.1 (C-1″), 97.2 (C-6), 96.1 (C-8), 79.2 (C-2), 77.6 (C-3″), 76.8 (C-5″), 73.6 (C-2″), 70.0 (C-4″), 61.1 (C-6″), 42.7 (C-3).

Dihydrokaempferol-7-O-β-D-glucopyranoside (**4**): White crystalline powder (MeOH), m.p. 152–154 °C, $[\alpha]_D^{25}$ −42.0° (c 0.2, MeOH), showing a dark blue coloration with ferric chloride reagent. Positive ion ESI-MS *m/z*: 451 $[M+H]^+$, 473 $[M+Na]^+$, 489 $[M+K]^+$; Negative ion ESI-MS *m/z*: 449 $[M-H]^-$; ^{1}H-NMR (400 MHz, CD_3OD) δ: 7.26 (2H, d, *J* = 8.2 Hz, 2′, 6′-H), 6.73 (2H, d, *J* = 8.2 Hz, 3′, 5′-H), 6.12 (1H, d, *J* = 2.0 Hz, 8-H), 6.10 (1H, d, *J* = 2.0 Hz, 6-H), 4.92 (1H, d, *J* = 11.6 Hz, 2-H), 4.86 (1H, d, *J* = 7.2 Hz, 1″-H), 4.50 (1H, d, *J* = 11.6 Hz, 3-H), 3.76 (1H, dd, *J* = 12.0, 1.6 Hz, 6″-Ha), 3.57 (1H, dd, *J* = 12.0, 5.2 Hz, 6″-Hb), 3.24–3.38 (4H, m, 2″-H~5″-H); ^{13}C-NMR (100 MHz, CD_3OD) δ: 199.3 (C-4), 167.2 (C-7), 164.7 (C-8a), 164.2 (C-5), 159.2 (C-4′), 130.3 (C-2′,6′), 128.9 (C-1′), 116.0 (C-3′,5′), 103.4 (C-4a), 101.1 (C-1″), 98.2 (C-6), 96.9 (C-8), 85.0 (C-2), 78.1 (C-3″), 77.6 (C-5″), 74.5 (C-2″), 73.7 (C-3), 71.0 (C-4″), 62.1 (C-6″).

Dihydroquercetin-7-O-β-D-glucopyranoside (**5**): White crystalline powder (MeOH), m.p. 151.0–153.0 °C, $[\alpha]_D^{25}$ −48.2° (c 0.2, MeOH), showing a dark blue coloration with ferric chloride reagent. Positive ion ESI-MS *m/z*: 467 $[M+H]^+$, 489 $[M+Na]^+$, 505 $[M+K]^+$; Negative ion ESI-MS *m/z*: 465 $[M-H]^-$; ^{1}H-NMR (400 MHz, CD_3OD) δ: 6.96 (1H, d, *J* = 2.0 Hz, 2′-H), 6.84 (1H, dd, *J* = 8.2, 2.0 Hz, 6′-H), 6.79 (1H, d, *J* = 8.2 Hz, 5′-H), 6.21 (1H, d, *J* = 2.2 Hz, 8-H), 6.19 (1H, d, *J* = 2.2Hz, 6-H), 4.55 (1H, d, *J* = 12.0 Hz, 3-H), 3.86 (1H, dd, *J* = 12.4, 2.0 Hz, 6″-Ha), 3.66 (1H, dd, *J* = 12.4, 5.6 Hz, 6″-Hb), 3.32–3.48 (4H, m, 2″-H~5″-H), 2-H and 1″-H were overlapped by water signal. ^{13}C-NMR (100 MHz, CD_3OD) δ: 199.4 (C-4), 167.4 (C-7), 164.8 (C-5), 164.3 (C-8a), 147.3 (C-4′), 146.4 (C-3′), 129.7 (C-1′), 121.1 (C-6′), 116.1 (C-5′), 116.0 (C-2′), 103.5 (C-4a), 101.3 (C-1″), 98.3 (C-6), 97.1 (C-8), 85.4 (C-2), 78.3 (C-3″), 73.9 (C-3), 77.8 (C-5″),74.7 (C-2″), 71.2 (C-4″), 62.3 (C-6″).

Quercetin-7-O-β-D-glucopyranoside (**6**): Yellow crystalline powder (MeOH), m.p. 173–175 °C, showing a dark blue coloration with ferric chloride reagent. Positive ion ESI-MS *m/z*: 465 $[M+H]^+$; Negative ion ESI-MS: 463 $[M-H]^-$; ^{1}H-NMR (400 MHz, CD_3OD) δ: 7.65 (1H, br s, 2′-H), 7.56 (1H, d, *J* = 8.2 Hz, 6′-H), 6.78 (1H, d, *J* = 8.2 Hz, 5′-H), 6.35 (1H, d, *J* = 2.0 Hz, 6-H), 6.62 (1H, d, *J* = 2.0 Hz, 8-H), 4.95 (1H, d, *J* = 7.2 Hz, 1″-H), 3.84 (1H, dd, *J* = 12.2, 1.6 Hz, 6″-Ha), 3.63 (1H, dd, *J* = 12.2, 5.6 Hz, 6″-Hb), 3.30–3.50 (4H, m, 2″-H~5″-H). ^{13}C-NMR (100 MHz, CD_3OD) δ: 177.4 (C-4), 164.4 (C-7), 162.1 (C-5), 157.6 (C-8a), 148.9 (C-2), 148.7 (C-3′), 146.2 (C-4′), 137.9 (C-3), 123.9 (C-1′), 121.8

(C-6′), 116.2 (C-2′), 116.1 (C-5′), 102.3 (C-4a), 101.6 (C-1″), 100.1 (C-6), 95.5 (C-8), 78.3 (C-3″), 77.8 (C-5″), 74.7 (C-2″), 71.2 (C-4″), 62.4 (C-6″).

Gambiriin A₃ (**7**): Light brown crystalline powder (MeOH), m.p. 172–174 °C, $[\alpha]_D^{25}$ −8.1° (c 0.5, Me_2CO), showing a dark blue coloration with ferric chloride reagent. Positive ion ESI-MS *m/z*: 581 $[M+H]^+$, 603 $[M+Na]^+$; Negative ion ESI-MS *m/z*: 579 $[M-H]^-$. ^{1}H-NMR (400 MHz, CD_3OD) δ: upper unit: 6.78 (1H, d, *J* = 8.4 Hz, 5′-H), 6.75 (1H, dd, *J* = 1.6, 6.8 Hz, 6′-H), 6.67 (1H, d, *J* = 2.0 Hz, 2′-H), 5.90 (2H, s, 3,5-H), 4.83 (1H, d, *J* = 3.2 Hz, *α*-H), 4.58 (1H, br s, *β*-H), 2.90 (1H, dd, *J* = 4.8, 14.8 Hz, γ-Ha), 2.50 (1H, dd, *J* = 10.0, 14.8 Hz, γ-Hb); terminal unit: 6.87 (1H, d, *J* = 1.2 Hz, 2′-H), 6.75 (1H, dd, *J* = 1.6, 6.8 Hz, 6′-H), 6.65 (1H, d, *J* = 8.0 Hz, 5′-H), 6.02 (1H, s, 8-H), 4.62 (1H, d, *J* = 7.6 Hz, 2-H), 4.03 (1H, m, 3-H), 2.90 (1H, dd, *J* = 4.8, 16.4 Hz, 4-Ha), 2.62 (1H, dd, *J* = 8.4, 16.4 Hz, 4-Hb). ^{13}C-NMR (100 MHz, CD_3OD) δ: upper unit: 158.2 (2C, C-2,6), 158.0 (C-4), 146.5 (C-4′), 144.3 (C-3′), 135.5 (C-1′), 120.8 (C-6′), 117.0 (C-2′), 116.2 (C-5′), 106.6 (C-1), 96.2 (2C, C-3,5), 78.1 (C-*β*), 46.6 (C-*α*), 29.5 (C-γ); terminal unit: 156.8 (C-7), 156.2 (C-5), 155.4 (C-8a), 146.5 (C-4′), 146.0 (C-3′), 132.6 (C-1′), 120.4 (C-6′), 116.4 (C-5′), 115.6 (C-2′), 108.5 (C-6), 102.1 (C-4a), 95.6 (C-8), 83.0 (C-2), 69.4 (C-3), 30.6 (C-4).

Gambiriin A₁ (**8**): Light brown crystalline powder (MeOH), m.p. 167–169 °C, $[\alpha]_D^{25}$ −12.7° (c 0.5, Me_2CO), showing a dark blue coloration with ferric chloride reagent. Positive ion ESI-MS *m/z*: 581 $[M+H]^+$, 603 $[M+Na]^+$; Negative ion ESI-MS *m/z*: 579 $[M-H]^-$. ^{1}H-NMR (400 MHz, CD_3OD) δ: upper unit: 6.75 (1H, br s, 2′-H), 6.63 (2H, br s, 5′, 6′-H), 5.84 (2H, s, 3,5-H), 4.70 (1H, br s, *α*-H), 4.59 (1H, br s, *β*-H), 2.90 (1H, m, γ-Ha), 2.49 (1H, m, γ-Hb); terminal unit: 6.78 (1H, d, *J* = 1.6 Hz, 2′-H), 6.68 (1H, d, *J* = 8.0 Hz, 5′-H), 6.63 (1H, br s, 6′-H), 6.04 (1H, s, 6-H), 4.70 (1H, br s, 2-H), 3.93 (1H, m, 3-H), 2.90 (1H, m, 4-Ha), 2.56 (1H, m, 4-Hb); ^{13}C-NMR (100 MHz, CD_3OD) δ: upper unit: 158.5 (2C, C-2, 6), 158.0 (C-4), 146.0 (C-3′), 144.5 (C-4′), 136.2 (C-1′), 121.4 (C-6′), 117.4 (C-2′), 116.3 (C-5′), 106.6 (C-1), 96.4 (2C, C-3, 5), 77.6 (C-*β*), 46.9 (C-*α*), 30.8 (C-γ); terminal unit: 156.3 (C-7), 156.2 (C-5), 155.5 (C-8a), 146.5 (C-3′), 146.4 (C-4′), 132.8 (C-1′), 120.8 (C-6′), 116.5 (C-5′), 115.6 (C-2′), 107.9 (C-8), 101.4 (C-4a), 97.9 (C-6), 83.2 (C-2), 69.3 (C-3), 29.6 (C-4).

Catechin (6′-8) catechin (**9**): Brown amorphous powder. $[\alpha]_D^{25}$ −126.7° (c 0.33, MeOH), showing a dark blue coloration with ferric chloride reagent. Positive ion ESI-MS *m/z*: 579 $[M+H]^+$, 596 $[M+NH_4]^+$; Negative ion ESI-MS *m/z*: 577 $[M-H]^-$. ^{1}H-NMR (400 MHz, CD_3OD) δ: upper unit: 6.82 (1H, s, 2′-H), 6.64 (1H, s, 5′-H), 5.90,5.82 (2H, d, *J* = 2.0 Hz, 6,8-H), 4.78 (1H, d, *J* = 6.0 Hz, 2-H), 4.01 (1H, m, 3-H), 2.66 (1H, dd, *J* = 4.8,16.0 Hz, 4-Ha), 2.42 (1H, dd, *J* = 5.8,16.0 Hz, 4-Hb); terminal unit: 6.71 (1H, d, *J* = 2.0 Hz, 2′-H), 6.70 (1H, d, *J* = 8.0 Hz, 5′-H), 6.59 (1H, dd, *J* = 2.0,8.0 Hz, 6′-H), 6.08 (1H, s, 6-H), 4.73 (1H, d, *J* = 6.0 Hz, 2-H), 3.97 (1H, m, 3-H), 2.73 (1H, dd, *J* = 5.2, 16.0 Hz, 4-Ha), 2.60 (1H, dd, *J* = 5.8,16.0 Hz, 4-Hb). ^{13}C-NMR (100 MHz, CD_3OD) δ: upper unit: 157.3, 156.7 (3C, C-5,7,8a), 145.5 (2C, C-3′, 4′), 131.6 (C-1′), 126.2 (C-6′), 119.6 (C-5′), 114.0 (C-2′), 100.1 (C-4a), 96.1,95.2 (2C, C-6,8), 79.9 (C-2), 67.6 (C-3), 26.3 (C-4); terminal unit: 156.4, 154.6, 153.0 (3C, C-5, 7, 8a), 145.6 (2C, C-3′, 4′), 132.2 (C-1′), 119.1 (C-6′), 115.7 (C-5′), 114.3 (C-2′), 108.0 (C-8), 100.5 (C-4a), 95.7 (C-6), 81.7 (C-2), 68.1 (C-3), 27.0 (C-4).

Table S1. 400 MHz ^{1}H NMR and 100 MHz ^{13}C NMR data of **1** in CD_3OD [a].

**Position**	**δ$_H$**	**δ$_C$**	**HMBC** (H→C)
Narigenin			
2	5.31 (1H, dd, *J* = 12.8, 2.8 Hz)	79.7	C-4, 1′, 2′, 6′
3	3.07 (1H, dd, *J* = 16.8, 12.8 Hz)<br>2.66 (1H, dd, *J* = 16.8, 2.8 Hz)	43.3	C-2, 4, 1′
4		197.3	
4a		102.9	
5		164.9	
6	5.90 (1H, d, *J* = 2.0 Hz)	96.6	C-4a, 5, 7, 8
7		167.6	
8	5.88 (1H, d, *J* = 2.0 Hz)	95.8	C-4a, 6, 7, 8a
8a		164.3	
1′		133.5	
2′	7.29 (1H, d, *J* = 8.4 Hz)	128.4	C-2, 1′, 3′, 4′, 6′
3′	7.06 (1H, d, *J* = 8.4 Hz)	117.1	C-1′, 4′, 5′
4′		158.5	
5′	7.06 (1H, d, *J* = 8.4 Hz)	117.1	C-1′, 3′, 4′
6′	7.29 (1H, d, *J* = 8.4 Hz)	128.4	C-2, 1′, 2′, 5′, 4′
glucosyl			
1″	4.91 (1H, d, *J* = 7.6 Hz)	101.5	C-4′
2″	3.49−3.54(1H, m)	74.3	C-1″, 3″
3″	3.49−3.54 (1H, m)	77.5	C-2″, 4″
4″	3.42 (1H, m)	71.5	C-5″
5″	3.78 (1H, m)	75.1	C-4″, 6″
6″	4.38 (1H, dd, *J* = 12.0, 8.4 Hz)<br>4.61 (1H, dd, *J* = 12.0, 2.0 Hz)	64.4	C-5″, 7‴
galloyl			
1‴		120.8	
2‴	7.10 (1H, s)	109.8	C-1‴, 3‴, 4‴, 6‴, 7‴
3‴		146.0	
4‴		139.4	
5‴		146.0	
6‴	7.10 (1H, s)	109.8	C-1‴, 2‴, 4‴, 5‴, 7‴
7‴ (C=O)		167.9	

[a] The δ$_H$ and δ$_C$ values were recorded using solvent signals (CD_3OD: δ$_H$ 3.31/δ$_C$ 49.0) as references. Signal assignments were based on the results of ^{1}H–^{1}H COSY, HMQC and HMBC experiments.

Figure S1. UV Spectrum of **1**.

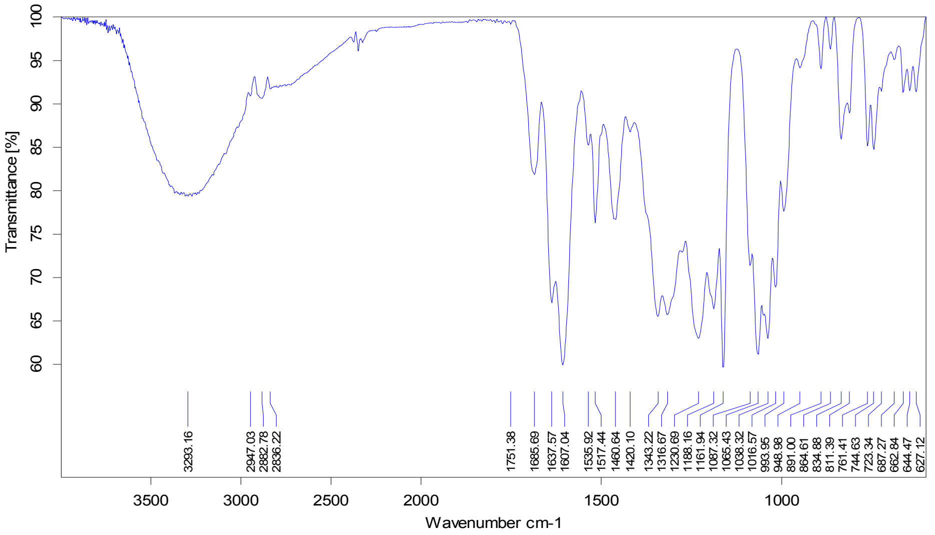

Figure S2. CD Spectrum of **1** in CH_3OH.

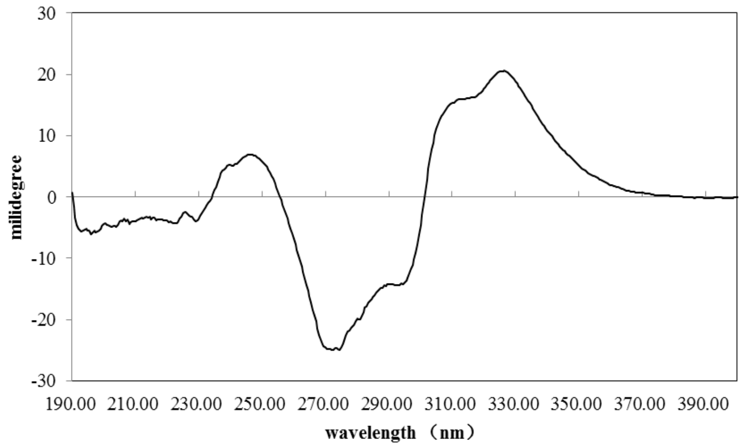

Figure S3. ESI-MS of **1**.

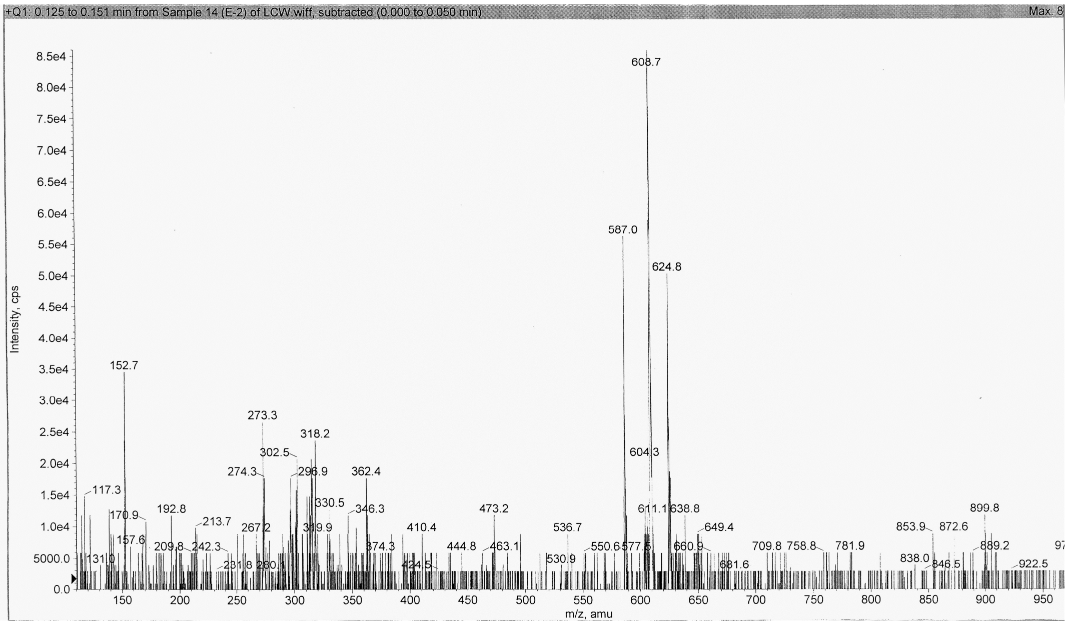

Figure S4. HR-ESI-MS of **1**.

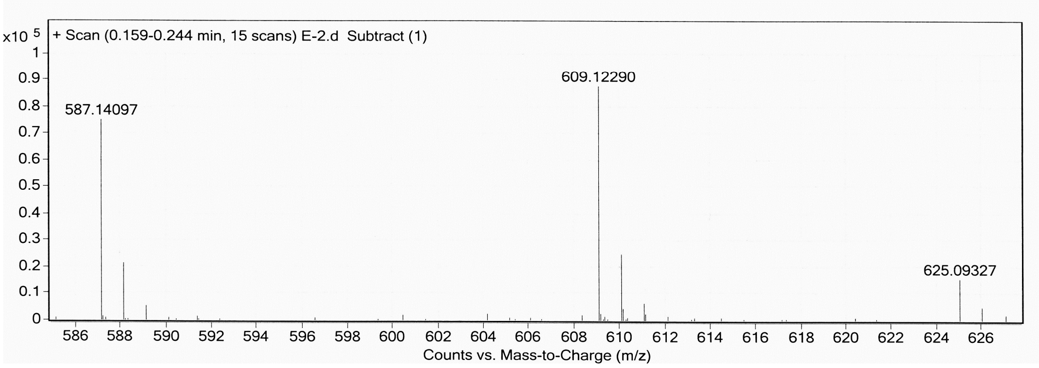

Figure S5. ^{1}H-NMR spectrum of **1** in CD_3OD.

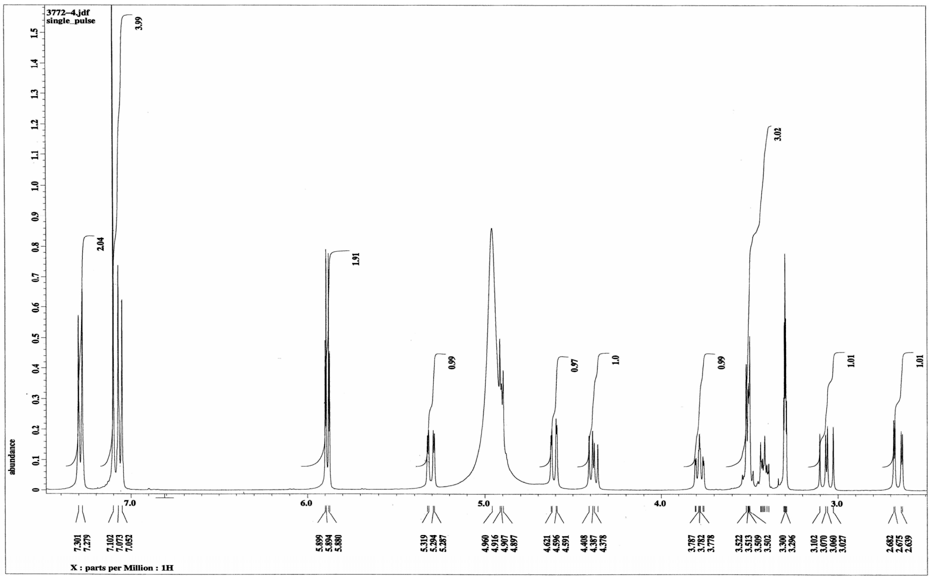

Figure S6. ^{13}C-NMR spectrum of **1** in CD_3OD.

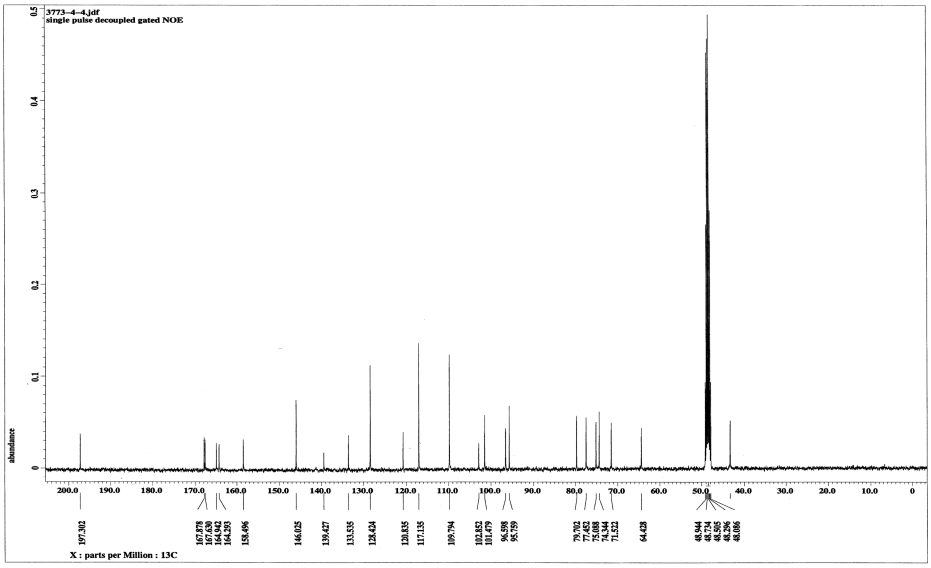

Figure S7. HMBC spectrum of **1** in CD_3OD.

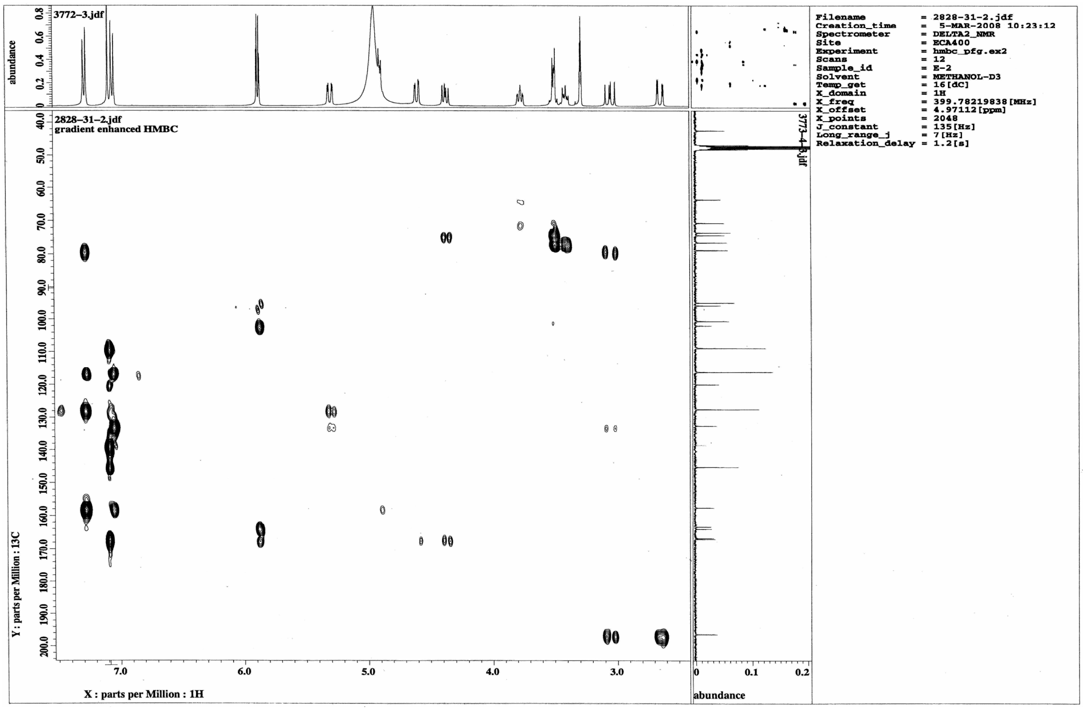

Supplement: Supplementary file 1 [file molecules-19-21363-s001.pdf]
